# The collaboration between Swiss initial vocational education and training partners: perceptions of apprentices, teachers, and in-company trainers

**DOI:** 10.1186/s40461-021-00114-2

**Published:** 2021-03-20

**Authors:** Florinda Sauli

**Affiliations:** Swiss Federal Institute for Vocational Education and Training, Av. de Longemalle 1, 1020 Renens, Switzerland

**Keywords:** Initial vocational education and training, Dual system, Partnership, Collaboration, Boundary crossing

## Abstract

This study aims to investigate the Swiss initial vocational education and training (IVET) partnership from the perspective of several stakeholders on the ground. Collaboration between stakeholders is essential in dual IVET to connect school- and workplace-based learning and to ensure the quality of the entire system. However, such collaboration can be challenging, given the different epistemic natures of the school and the training company. Apprentices, that regularly cross the boundaries of vocational school and training company, often struggle to connect the learnings that they have acquired from both places. Adopting a boundary crossing perspective, we explore perceptions of IVET partnership in terms of challenges and learning opportunities for the stakeholders on the ground. We realized focus groups with apprentices, vocational teachers, and in-company trainers (N = 64) from several professional fields. The data were analyzed in an inductive and deductive manner, using a thematic analysis. The main results highlight that the participants consider the collaboration between stakeholders to be weak: the links between schools and training companies appear to be scarce and not supported by explicit or formal strategies. Further, the apprentices act as brokers, but they are often not supported in connecting school- and workplace-based learning. These results can provide new insights into how the IVET partnership could be designed.

## Introduction

In initial vocational education and training (IVET) “dual systems” combine school- and work-based learning. According to certain scholars, this arrangement results in a better integration of apprentices into the working world, high-quality occupational skills, and low levels of youth unemployment (Busemeyer and Trampusch 2012; Gonon 2017). A dual system implies the existence of two learning locations—vocational schools and training companies—each with their own rationalities and rules (Gurtner et al. 2012; Nieuwenhuis and Woerkom 2007). During their training, apprentices regularly cross the boundaries between school and workplace; in order to learn the trade, they must connect knowledge from both places (Guile and Young 2003). Such dual systems, in which both the state (public sector) and the company (public and private sector) are responsible for the provision of IVET are considered in political economy as “collective skill formation systems” (Busemeyer and Trampusch 2012). In such systems, the several stakeholders involved—the state, schools, and companies—are collectively organized and strongly dependent on one another. Their collaboration is therefore not a matter of choice but necessity (Emmenegger et al. 2019). This collaboration—or IVET partnership—is seen as a precondition for the success and quality of the IVET system (Euler 2004). However, such collaboration can be challenging because the partners involved may hold different views on learning and have different cultures and priorities (Tynjälä 2008). For example, in vocational schools, the focus is on learning, while, in companies, it is on productivity (Gurtner et al. 2018).

In Swiss IVET, the collaboration between learning locations is enshrined in law, and formal rules have been established to ensure that it takes place (The Federal Assembly of the Swiss Confederation 2002). However, a gap exists between formal rules on one side, and beliefs and actual practices on the other (Emmenegger et al. 2019). For example, the rules could be established in a loose way, leaving space for interpretation, and resulting in the stakeholders employing heterogeneous practices (Aprea and Sappa 2015). Stakeholders also share different conceptions about how learning locations should be connected, which impacts their collaboration (Sappa et al. 2018). Therefore, a top-down approach that analyzes IVET collaboration solely from the perspective of what is established by the law is incomplete.

Thus, our study aimed at investigating the perception of IVET partnership from the point of view of three stakeholders on the ground: apprentices, vocational teachers, and in-company trainers. Through their interaction and collaboration, these stakeholders participate in shaping the entire IVET system (Emmenegger et al. 2019), demonstrating the extent to which their role is pivotal. Some scholars have highlighted how certain reforms in IVET educational policies led to the inclusion of collaboration and networking in the daily agenda of vocational teachers and in-company trainers (Sappa et al. 2018; Tynjälä and Nikkanen 2009). As we will see throughout the study, apprentices can be considered brokers between the school and the workplace—or intermediaries between teachers and in-company trainers—therefore exerting an influence on them (Akkerman and Bakker 2012).

We adopt a boundary crossing approach because it focuses on those transitions between contexts that are socioculturally different. Indeed, this approach has already been used in the field of dual IVET to understand how apprentices can be helped in establishing connections between their practices at the vocational school and those at the training company (for example, see Akkerman and Bakker 2012; Caruso et al. 2020; Flynn et al. 2016; Sappa et al. 2018; Tanggaard 2007). This approach is therefore relevant for studying IVET partnerships wherein stakeholders from several intersecting practices (state, school, training company) must collaborate with each other despite their sociocultural differences. The literature emphasizes that, in an IVET partnership, stakeholders can share mutual benefits (Billett and Seddon 2004; Flynn et al. 2016). Moreover, collaboration can trigger processes of mutual understanding and learning for all the stakeholders involved (Akkerman and Bruining 2016; Sappa et al. 2018). This can improve the connection between school and training company.

## Conceptual framework

In this section, we first introduce the approach of boundary crossing and explain how it can generate learning processes through collaboration. Second, we focus on the implications of IVET partnership in the connection of school- and workplace-based learning in general and, finally, in the context of Swiss IVET.

### Boundary crossing to foster the collaboration between school and workplace

“All learning involves boundaries,” is how Akkerman and Bakker (2011, p. 132) start their literature review titled “Boundary crossing and boundary objects.” As per their definition, a boundary is double-faceted: “[it] can be seen as a sociocultural difference leading to discontinuity in action or interaction” but also “[…] simultaneously suggest a sameness and continuity in the sense that within discontinuity two or more sites are relevant to one another in a particular way” (Akkerman and Bakker 2011, p. 133). The concept of boundary is particularly relevant in a setting such as that of dual IVET, wherein apprentices regularly cross the boundaries between school and workplace. Not only apprentices but also teachers and in-company trainers must face, as a part of their everyday practices, the challenges posed by the sociocultural differences that exist between these two learning locations. They must also determine ways to reconcile these experiences. Boundary crossing refers to the transitions and interactions of individuals across sites (Suchman 1994), and it implies that action and interaction should be re-established between the two spheres despite their differences. In this context, boundaries are not seen as barriers but as potential learning resources. It is specifically the ambiguity that boundaries generate—belonging either, or neither, to both domains—that triggers the need to redefine meanings and produce new practices and learning (Akkerman and Bakker 2011). Objects can also fulfil this function of bridging several sites, in which case we refer to “boundary objects” (Star 1989).

How can boundaries generate learning? Akkerman and Bakker (2011) distinguished four learning mechanisms that can occur in IVET partnerships (Akkerman and Bruining 2016): identification (i.e., defining how practices are different from one another and how they can coexist), coordination (i.e., finding effective means through which practices can cooperate with minimal interaction), reflection (i.e., redefining one’s practice by adopting another’s perspective), and transformation (i.e., collaborating to adjust current practices or defining new ones to solve a common problem). In addition, boundary crossing can occur at different levels (Akkerman and Bruining 2016): institutional (between multiple organizations; e.g., vocational schools and training companies), interpersonal (between specific groups of people from different practices; e.g., a group of teachers and a group of in-company trainers), and intrapersonal (when a person participates in several practices; e.g., an apprentice who transitions between school and training company). In this latter case, we speak about “brokers” (or “boundary crossers”; Akkerman and Bruining 2016). Brokers hold a powerful, but ambiguous, position because they “can either use information to retain positional authority and their status as a link between two or more groups or use it to join people together for mutual benefit” (Akkerman and Bruining 2016, p. 250). Griffiths and Guile (2004) pointed out that apprentices should be assisted by teachers and in-company trainers to grow as brokers. This can foster the connection between the school and the workplace, and lead teachers and in-company trainers acting as “boundary crosser facilitators” (Sappa et al. 2018). The literature on school-workplace connectivity provides a theoretical framework that supports a systemic approach to connect school and workplace. It emphasizes that the stakeholders must develop a shared understanding of their respective roles and responsibilities with regard to supporting students’ learning (Billett and Seddon 2004; Griffiths and Guile 2004; Tynjälä 2008). Such an understanding can be fostered through, for instance, regular contact between stakeholders, maintaining a benevolent attitude while communicating and collaborating, and promoting hybrid profiles (e.g., teachers who have experience of the trade, or in-company trainers with pedagogical skills; Onstenk and Blokhuis 2007; Sappa et al. 2018; Tynjälä 2008).

### The necessary collaboration to integrate school- and workplace-based learning

In many dual IVET systems (e.g., Danish, German, or Swiss), practices for connecting school- and workplace-based learning are well established. Nevertheless, certain studies revealed a lack of agreement between teachers and in-company trainers regarding their functions and perceptions with respect to collaboration (Deitmer and Heinemann^2009^; Sappa and Aprea 2014; Tynjälä 2008; Wesselink et al. 2010). In the German dual apprenticeship system, Gessler (2017) found that companies and vocational schools were “loosely coupled with a dominant partner (i.e., companies) and a subordinate partner (i.e., vocational schools)” (p. 164). Specifically, companies do not always collaborate and coordinate their work with the schools. Tynjälä (2008) explained that the training companies expect teachers to initiate the collaboration because “educational matters are the primary responsibility of educational institutions and only of secondary interest to workplaces […]” (p. 149). Further, teachers are considered responsible for aligning the content of the school curricula with that of the training company (Sauli et al. 2021).

In the literature, we can find some attempts to categorize the partnership between IVET stakeholders. This include Euler’s (2004) cooperation between learning locations (*Lernortkooperation*) and Gessler’s (2017) work on the collaboration between companies and schools in the German dual apprenticeship system. Both these categorizations introduce three levels of collaboration between schools and training companies. The first level usually consists of an implicit agreement, or a simple exchange of information, in which schools and training companies fulfil their respective duties. In the second level, the degree of interdependence between the schools and the training companies is higher. It entails a certain agreement regarding the respective roles and responsibilities related to implementing the established measures. In the context of boundary crossing, this level can be associated with the learning mechanisms of identification and cooperation. Finally, in the third level, schools and training companies work together directly with the intention of yielding mutual benefit (boundary crossing’s learning mechanisms of reflection and transformation). The three levels have to be seen as a linear sequence: Joint activities would be impossible without an exchange of information (Peter 2014). Moreover, a further division can be introduced between “compulsory collaboration” and “desirable collaboration” (Peter 2014). Compulsory collaboration should be required by law to ensure a minimum exchange between the two parties (e.g., in terms of the exchange of information and the efforts required to coordinate curricula). All further forms of collaboration (e.g., joint training projects) are “desirable” in that, while they are recommended and welcomed, they must be fulfilled on a voluntary basis. Certain aspects of collaboration cannot be regulated (e.g., the full coordination of learning sequences between learning sites), because this would render the system excessively inflexible (Peter 2014).

The two categorizations of IVET partnerships share certain similarities and differences. To increase the level of collaboration, both models stress the importance of triggering a change in the behavior of schools and training companies. In Euler’s (2004) model, the focus is on information exchange that can lead to an adjustment in behavior based on the new information received. As per the work of Gessler (2017), increased collaboration can be triggered either by unexpected events and problems (“corrective co-operation,” which is problem-driven and past-oriented) or the anticipation of unintended events (“expansive co-operation,” which is goal-driven and future-oriented). In both categorizations, in the highest level of collaboration, there is creation of a “third space” through the joint activities between schools and training companies. This hybrid space allows for the co-construction and establishment of new practices and the development of learning. Many sociocultural approaches to learning at the community, organization, or network level include the idea of a hybrid third space. In this space, there is an overlap of the several worlds involved and the creation of new practices and learning (Tynjälä 2008). According to the theory of expansive learning (Engeström 1987), stakeholders cannot face the challenges of the integration of school- and workplace-based learning alone. They can only be overcome through the establishment of interaction and collaboration between the stakeholders: “In expansive learning, learners learn something that is not yet there. In other words, the learners construct a new object and concept for their collective activity and implement this new object and concept in practice” (Engeström and Sannino 2010, p. 2). The focus is not on teaching and on the professionals who are in charge of said teaching but, rather, on learning with the understanding that all the stakeholders involved can learn something (*expanded* learning; Engeström and Sannino 2010). This means that not only apprentices, but also teachers and in-company trainers can learn. Finally, the influence that apprentices can have on the collaboration between schools and workplaces has not been adequately considered in the categorizations by Euler (2004) and Gessler (2017). This is despite the fact that apprentices play the crucial role of brokers.

### The collaboration between IVET stakeholders in the Swiss dual system

In Switzerland, scholars from several disciplines have investigated the partnership among IVET stakeholders (Emmenegger et al. 2019; Euler 2004; Gonon 2013; Sappa et al. 2018; Strebel et al. 2019). The collaboration between stakeholders has been formally mentioned in the Federal Act on Vocational and Professional Education and Training (VET/PET; The Federal Assembly of the Swiss Confederation 2002). This act defines the shared responsibilities of vocational schools, training companies, and branch course providers with respect to supporting student learning. Branch courses^1^ are a third learning place, which aims to complement workplace training and classroom instruction. The three learning locations are “explicitly required to interact and collaborate for curricula development, training implementation and assessment processes” (Sappa et al. 2018, p. 307). Compared to previous arrangements, the Federal Act on VET/PET of 2002 strengthened the collaboration between learning locations; it also created the legal basis for more flexible forms of such collaboration (Dubs 2004; Gonon 2018).

The learning aims and the roles of vocational schools and training companies are defined by law in a common curriculum (Aprea and Sappa 2015). IVET ordinances (i.e., training regulations) envisage a learning and performance documentation (LPD), a tool that an apprentice should use to take notes regarding the major tasks and experiences encountered at the workplace. They can also use LPD to record the skills they obtain, which can then be periodically checked by their in-company trainer (see for example, retail’s ordinance, State Secretariat for Education, Research and Innovation [SERI] 2004). The LPD was found to increase apprentices’ awareness about the connection between school and training company. It was also found to be useful for aligning the content covered by the learning locations (Caruso et al. 2016). Even though the LPD is supposed to be used only within the training company, its characteristics designate it as a tool for connecting school- and workplace-based learning. For this reason, some studies have explored the potential of the LPD as a boundary object (Caruso et al. 2016, 2020).

Despite these formal arrangements, the forms that the Swiss IVET partnership can take remain undefined and can therefore largely vary (Dubs 2004; Sappa and Aprea 2014). Studies in the IVET context reported that there exist certain issues between schools and training companies regarding collaboration. In particular, there are issues with helping apprentices connect the experiences they have gained from school and training company and aligning the content learned in the two locations (Gurtner et al. 2018; Sappa and Aprea 2014; Peter 2014). Further, Sappa and Aprea (2014) identified four conceptions about the connection across different learning locations in Swiss IVET that each influence the collaboration between stakeholders differently. According to them, the connections between learning locations can be classified as: (1) separate learning experiences (school, company and branch courses involve completely separate settings, each governed by its own standards); (2) complementary learning experiences (the school transmits conceptual knowledge that is applied at the training company); (3) mediation by a branch course center (school and training company are connected through branch courses); (4) school-centred integration (there is a circular and iterative integration process across the three learning locations). With regard to the IVET partnership, in the first and second conception, there is no expectation of collaboration between the learning locations, and the apprentices are deemed responsible to establish connections by themselves. In conceptions three and four, the level of collaboration between the learning locations increases, and the apprentices are supported by teachers, in-company trainers, or branch courses trainers to connect practices. Therefore, according to the conception of a person, they will have different expectations from the IVET partnership.

### Aims and research questions

The operation of the Swiss dual IVET is based on collaboration between decentralized stakeholders who need to connect practices from school and training company. Significant efforts have been made to promote and support this collaboration. However, the stakeholders on the ground, sometimes, experience a disconnection between learning locations. The aim of this study is to investigate the perspectives of apprentices, teachers, and in-company trainers regarding the IVET partnership as well as to determine ways to improve the same. Accordingly, the research questions have been defined as follows: (1) How do apprentices, teachers, and in-company trainers describe the partnership between IVET stakeholders? (2) Which elements of boundary crossing that emerge in the stakeholders’ discourses could improve the IVET partnership?

## Methods

This research was a part of a bigger project^2^ that included several studies that used both quantitative and qualitative methods. It focused on IVET quality from the point of view of apprentices, teachers, and in-company trainers. The IVET quality encompassed several elements that emerged spontaneously in the stakeholders’ perceptions, including IVET partnership. We therefore decided to more closely analyze the qualitative data on this topic.

### Participants

We conducted fourteen focus groups in French-speaking Switzerland with a total of 64 participants. These included groups of apprentices (n = 32; M_age_ = 20.75 years), teachers (n = 17; M_age_ = 46.6 years), and in-company trainers (n = 15; M_age_ = 44.7 years). Further, the participants were from one of four professional fields: (a) hair dressing and beauty; (b) commercial employee; (c) construction (e.g., carpenter, electrician); and (d) retail. The groups were uniform in terms of stakeholders and professional fields. They were composed of three to seven participants, which allowed each participant to express their views and ensured a smooth discussion. The participants were selected according to their role (apprentices, teachers or in-company trainers) and professional field. During the selection process, care was taken to ensure a certain heterogeneity within the groups (e.g., apprentices from different school years, teachers of different subjects, trainers from different companies). Finally, we also targeted different geographical areas. Each focus groups started with three participants, and selection was stopped when the maximum number per group (seven participants) was reached. Participation in the study was voluntary.

### Data collection

The research project of which this study was a part of aimed to identify elements associated with IVET quality from the point of view of multiple stakeholders. Therefore, we opted for a data collection technique that enable the perceptions of IVET quality to emerge spontaneously: focus groups (Duchesne and Haegel 2004; Kitzinger 1995; Krueger 2002). This method consists of simultaneously interviewing several people who have certain experiences and/or professional affiliations in common. The method favors the analysis of attitudes, beliefs, or norms shared by a particular group. The interviews were carried out in French by the research team and were semi-directive. The interview framework used was developed by the research team and based on the pilot study of the research (Sauli et al. in press). We opted to employ open-ended questions regarding the perceived positive aspects and gaps of IVET (e.g., “Can you mention some positive points of your training at school supported by examples?”) to let themes emerge spontaneously in participants’ discourses. The focus was more on the training at the vocational school and at the training company, than on branch courses. During the interviews, the participants were encouraged by the research team to illustrate their thoughts using concrete examples. Ultimately, the major themes that emerged during the discussion were summarized on the whiteboard, and the participants were allowed to check, add, or modify the material. The interviews, which, on average, went on for an hour, were recorded, transcribed, and anonymized.

### Data analysis

The level was selected based on the group and not the individuals in it. Accordingly, the results are to be understood as reflective of the collective perceptions of the IVET stakeholders. The transcriptions were analyzed using a thematic analysis (Krippendorff 2013; Miles and Huberman 1994; Saldaña 2013) with a hybrid approach of deductive and inductive coding (Fereday and Muir-Cochrane 2006). First, an inductive analysis of the data was conducted with a focus on retaining as much of the original meaning of the responses as possible. Second, broader categories and links between them were established based on existing theories. During the coding process, discussions were conducted regularly among the members of the research group to reduce interpretation bias. The coding scheme was refined over several rounds to reduce the overlapping of codes. A codebook was developed, which contained the labels, definitions, descriptions, and inclusion and exclusion criteria of the codes. Ultimately, 17 codes emerged as central aspects related to IVET quality. Only the code *Contact between learning locations*^3^ (n = 40 coding units) is analyzed in this paper. In addition, it was also subjected to a further round of thematic analysis, which followed the same procedure that was explained previously.

To answer our research questions, we analyzed the data through the perspective of apprentices, teachers, and in-company trainers. The differences in terms of professional fields were not relevant in our sample, and, therefore, the associated data has not been provided here.

## Results^4^

The analysis of the focus groups interviews revealed five main themes in IVET partnership according to the perspectives of apprentices, teachers and in-company trainers (see Appendix for more information). In the following sections, the main themes are described individually.

### Degree of formality in an IVET partnership

In their discourses about contact between stakeholders, apprentices, teachers and in-company trainers highlighted a lack of both formal and informal contact.

- *Formal contact* is governed by regulations or prearranged agreements between stakeholders. The apprentices hold varied opinions regarding formal contact, as demonstrated by the following exchange between apprentices from the hairdressing field:
  *Apprentice 1*: *If we miss class, our in-company trainers get an email directly from the school that informs them […]*
  *Interviewer*: * Do you think it’s good to have such links?*
  *Apprentice 2*: *Yes, because I think there would be a lot of abuse if it wasn't for that.*
  *Apprentice 3*: *Yes, there would be too much abuse.*
  *Apprentice 1*: *I don’t think it’s good. That way, we can’t skip school.*
  *Interviewer*: *And the marks? You must submit them to your in-company trainers?*
  *Apprentice 2*: *Not necessarily.*
  *Apprentice 1*: *It depends on the trainers.*
  *Apprentice 3*: *Every semester, they automatically receive the marks […]. I think it’s good because it maintains a link [between school and training company][…]. At least, there is something there. You just have to be careful not to go down again. […], it gives a little… Reinforcement, just in case.*

Certain apprentices believed that it is good to have formal measures to govern the transmission of information, as it alleviates their responsibility, prevents abuse, and allows them to receive supervision. For others, these formal measures are excessively restrictive, as they do not allow apprentices enough autonomy. Teachers and in-company trainers seem to favor formal contact—and they would like to have even more of it - as far as it does not entail too much administrative work.

- *Informal contact* is not defined by regulations. Instead, it arises from initiatives taken by a person or organization. Since such contact is generally personal, on one hand, it can be motivated by authentic interest and good intentions. On the other hand, it is very much dependent on the stakeholders’ level of engagement and can also easily collapse if the people involved change over time. This dynamic is described in the following statement. A network between vocational schools, training companies, and parents was initiated but eventually abandoned following certain changes to the network:
  *[…] in terms of communication, once a year, the vocational school welcomed parents and companies to present the learning process. That was good, what they were doing at the time […] I like to plan the school year before welcoming the apprentice […] Some schools, when you phone in mid-July, have no problem in passing on their schedule, while others respond with “No, please wait until the start of the next school year and your apprentice will give it to you.” (Commercial Employees In-Company Trainer).*

Moreover, it seems that contact occurs only if there are problems with an apprentice, such as absences, low grades, or personal issues:

*[…] We make contact when things start to go wrong […] In general, if the apprentice is operating normally and is in the right standard, we have very, very little contact with in-company trainers (Construction Teacher).*

Such contact can create the basis for higher collaboration between stakeholders:

*In my experience, I’ve noticed that if I have problems with students in class, it’s often the case at the training company as well. […] That’s what also creates this bond with the in-company trainer. We call and tell him, “Well, we’re having problems with your apprentice, there’s this, there’s that.” He says, “Yeah, me too.” Many times, it’s like this (Hairdressing Teacher).*

Informal contact is a topic raised mainly by teachers and in-company trainers. Both these groups are uncertain about who should initiate contact. The majority think that it is the others’ responsibility to do it. Some of them do not initiate contact because they do not want to interfere with their apprentices’ lives. They also believe that assuming a “maternal/paternal” attitude toward their apprentices propagate an incoherent message about the independency that these youngsters should develop to be successful in their trade. This aspect has been illustrated by commercial employees’ in-company trainers:

*In-Company Trainer 1*: *I wouldn’t find it easy to get in touch with schools because there is also respect for the young person’s life. I also don’t feel I have the right to interfere if it’s not necessary.*
*In-Company Trainer 2*: * […] I find that, sometimes, both the school and, perhaps, some counsellors are still very much playing the role of mum or dad, whereas we should be more focused on coaching the young person to help them effectively utilize their resources […] and I also think that the message given to these young people is incoherent with what is expected of them in the workplace.*

### Apprentices as brokers

Another theme that emerged in teachers’ and in-company trainers’ discourses was apprentices’ potential for navigating the transmission of information from one learning location to another. This was especially relevant if there was no explicit agreement among stakeholders regarding their collaboration. The apprentices’ room for manoeuvre was seen either in a negative or positive way, depending on different stakeholders. Any negative perspectives found were mainly held by in-company trainers. Some of them complained that they only received feedback from the school through apprentices. In the absence of the appropriate protocols, the comprehensiveness of the transmitted information, along with its accuracy and transparency, remains under the control of the apprentices. Certain trainers expressed doubts regarding the “good faith” of certain apprentices in relation to the transmission of information:

*It’s not clear to us exactly what they’re doing. We don’t receive any feedback from the school, and it is our apprentices who come and give us the information that may not be correct (Retail In-Company Trainer).*

Some teachers hold a more positive perspective and mentioned being able to stay updated with the trends being employed at the workplace through their apprentices (e.g., fashionable haircuts in the hairdressing industry). Teachers are also able to make companies aware of certain important topics related to the trade, which are not necessarily being practiced by everyone, such as the safety standards implemented in the workplace:

*Through apprentices, we can orient and, perhaps, sensitize the training company. For example, we are now moving more and more toward health protection in terms of gloves, and it’s true that some hairdressing salons are having trouble getting started. But at the same time, we are making them aware of the fact that wearing gloves is important (Hairdressing Teacher).*

### Teachers and in-company trainers as brokers

Participants often mentioned situations in which teachers or in-company trainers play more than one role. This implies that they contribute to more than one learning location (for example, a retail teacher who also works in a shop and trains apprentices) or have relevant previous experiences from other learning locations. Such hybrid profiles are considered a good practice by all participants. For teachers, working in the field seemed important to remain updated with the evolution of the trade and gain credibility in the eyes of their apprentices. This can be explained by the following exchange between hairdressing teachers:

*Hairdressing Teacher 1*: *[…] I think it’s important because, as a teacher you can become a person who “just talks.”*
*Hairdressing Teacher 2*: * It disconnects you a bit.*
*Hairdressing Teacher 1*: * With the theory, you are completely disconnected from what is going on at the hairdressing salon. While there [at the hairdressing salon], at least, I’m working.*
*Hairdressing Teacher 2*: * We are more credible too, to apprentices […].*
*Hairdressing Teacher 1*: * That’s also how we see the evolution in the salon.*

The experience gained from the company can also be useful if transferred to school (and vice-versa):

*The positive side is that we have professionals who work in offices, who see what is happening in offices, and who can bring this knowledge to school while maintaining a global vision (Construction Teacher).*

### Boundary objects and other brokers

In the discourses of apprentices, teachers and in-company trainers, we identified several elements that play the role of boundary objects or brokers:

- *Branch courses:* Apprentices expressed mixed opinions about branch courses. Some considered them useful and appreciated the advantage of not having all the constraints of a school (schedule, homework, tests, etc.). Others consider the courses useless if they fail to align with the other learning locations in terms of content or learning sequences, or if they are overly repetitive. Further, certain apprentices experience difficulties with reconciling the demands (homework, tests, etc.) and requirements put forward by the three learning locations.
- *Learning and performance documentation (LPD):* Apprentices considered the LPD useful because it comprises the topics of the exams and allows for self-evaluations of the relevant skills. It also allows the structuring of the training accordingly, as explained in the following extract:
  *[…] It helps us to determine how to manage our training, where we are. It’s more like objectives that will help us know what we have to do and then... yeah, improve ourselves […]. We have tasks to complete, and, until we’ve not passed them, we can’t validate our documentation (Retail Apprentice).*

According to this extract, the LPD fulfils several functions (Caruso et al. 2016): monitoring, evaluation, reference, and planning. Namely, it can help monitor the learning process and the (self-)assessment of the learners’ competence development. As for the negative aspects of the LPD, the apprentices referred to a misalignment or a repetition of the matter taught at school. They also highlighted the fact that the practices associated with LPD are extremely heterogeneous and have not been formally defined. The use and control of the LPD largely depends on in-company trainers or the employer’s training organization. Apprentices also complained that there was no designated time for filling out the LPD. This means that it has to be completed outside of the training hours, during their personal time.

- *Apprenticeship inspectors*^5^ can fulfil the role of brokers. For certain teachers, apprenticeship inspectors are required to ensure that a connection between the school and the workplace is established, even though this is not officially their responsibility. The inspectors tend to ensure a connection between learning locations only when there is a problem with an apprentice. According to a construction teacher, the training inspectors are key actors who are required to monitor the alignment between school- and work-based learning. For example, they can do this by checking in with the apprentice and the in-company trainer every 6 months.
- *Practical courses at vocational school*: For apprentices they should be more aligned with the workplace or with exams; more time should be devoted to them, while less time should be dedicated to general culture classes. Further, teachers should also use more visual representations and authentic objects to make the education provided more similar to that provided at the workplace.

### Examples of networks

The teachers and in-company trainers provided some examples of networks (as in the third level of collaboration in the models of Euler 2004 or Gessler 2017). Such networks seemed to arise more easily in relatively small regions (with low population density) that had a communitarian background and in which “people know each other personally”. This was illustrated by this hairdresser teacher:

*[…] In our state, we’re really a team in everything. We have a lot of great projects with the employers, with the hairdressers, and with the teachers’ association. In the state of [name of the state], it’s not like everyone who has their own little role. We're really one – we’re all involved in everything. So it’s really nice, yes. We meet everyone, actually (Hairdressing Teacher).*

Often, networks are already established, for instance, by professional associations and they are active with regard to several initiatives, such as the organization of informal meetings between different stakeholders. Furthermore, sometimes a network can be created by a single highly committed person who takes the initiative to contact other stakeholders. According to our data, personal contact and dialogue seem to be the most effective ways of promoting relationships. Certain teachers and in-company trainers have an integrative vision for IVET partnerships and how the training should be carried out. They consider IVET comprehensive and the result of a joint effort. They act accordingly, as demonstrated by the following statement:

*[…] We don’t make any distinctions, such as this is the company, this is the school, these are the branch courses… For us, it’s a global training; and we are partners, we are together. I see it this way (Construction In-Company Trainer).*

Another example of an integrative vision of IVET can result from the mixing of novice and experienced professionals:

*[…] For the last three years, we’ve been working a lot as a network, as a group. So, we are in the process of creating communities of practice between trainers and apprentices. The idea is to be able to really put trainers together so that they can, based on their experiences, be included in this notion of co-development. Thus, we have new trainers, and we have those who are very experienced, who have 15 years of practice, who are also experts in examinations, or even apprenticeship inspectors; and, as a result, by putting them together, the novices benefit from the experience of the older professionals, while the older ones are also made to question their practices. […] We organize the same thing for apprentices, which means that the apprentices are also mixed […] (Commercial Employees’ In-Company Trainer).*

## Discussion

The aim of this study was to investigate how apprentices, teachers, and in-company trainers describe the IVET partnership and determine which elements of boundary crossing that emerge in their discourses could help improve this partnership. The results of this study indicate that five main themes can be identified: degree of formality in IVET partnerships, apprentices as brokers, teachers and in-company trainers as brokers, boundary objects and other brokers, and examples of networks.

The description of the IVET partnership (research question 1) is often dependent on the unidirectional exchanges of information between stakeholders that pass through the filters of one central stakeholder (apprentice) who acts as a broker. The formal measures that could help foster connections between stakeholders are seen in an ambivalent manner: Either they are not directive enough, or they are overly restrictive. Informal measures can be effective but are not easy to establish. The perspectives of the participants of this study regarding partnership can be associated with the first level of collaboration from the model of Gessler (2017). In said model, contact is only established in case of problems. Unsatisfactory performance and misbehavior by students are often the trigger for collaboration between the stakeholders (Peter 2014; Aprea and Sappa 2015). This results in what is referred to as “corrective coordination” in the literature, which could eventually lead to the more ideal “expansive coordination” (Gessler 2017). However, collaboration should not only focus on the apprentices’ problems, but also be a component of the daily responsibilities of the teachers and in-company trainers (Tynjälä and Nikkanen 2009). The results of the study also reveal that stakeholders have different conceptions regarding what the IVET partnership should be like, as found in the framework of Sappa et al. (2018). For certain stakeholders, learning locations are mostly separate worlds, and it is considered the responsibility of the apprentices to connect them. In contrast, others hold a more integrative view and tend to create networks based on personal contacts and the organization of joint activities. This was made evident by this previously cited excerpt: “*Some schools, when you phone in mid-July, have no problem passing on their schedule, while others say, “No, please wait until the start of the next school year and your apprentice will give it to you.”*

According to our analysis, several elements and levels of boundary crossing could be identified (research question 2). Apprentices, teachers, and in-company trainers can all play the role of brokers (intrapersonal level of boundary crossing; Akkerman and Bruining 2016). With regard to apprentices playing this role, there seem to be more problems in terms of exchanging information, taking responsibility, and having support to undertake this duty. The role of the apprentices as brokers appears to be extremely ambiguous and not completely acknowledged by the other stakeholders. In contrast, the teachers and in-company trainers who play the role of brokers are positively perceived and even welcomed. Brokers hold an ambiguous position that can also allow opportunities for introducing elements of one practice in another (Akkerman and Bakker 2012) and end up in the creation of expanded learning (Engeström 1987). This can be observed in our data. Learning is expanded through apprentices who cross the boundaries between school and workplace. There is “contamination” in both learning locations: At school, teachers can stay updated with the current needs of the trade, whereas, in the workplace, in-company trainers can be familiarized with the standards and best practices taken from a more academic milieu. It is the whole network that learns, and the focus is not on teaching but on learning (Engeström and Sannino 2010). Further, this example illustrates the potential for learning that exists at the boundaries. It is unrealistic to expect teachers or in-company trainers to consistently stay updated with the rapid developments that occur in a specific trade or scientific field and have both advanced pedagogical and vocational expertise. Therefore, by using the direct experiences of their apprentices, teachers or in-company trainers from different learning locations can inform one another (Bakker and Akkerman 2019). This can also occur in boundary crossing situations at the interpersonal and institutional levels through the creation of networks between stakeholders. An example of this could be the exchange of experiences between experienced and novice trainers and apprentices. This is also a good example of the learning mechanism of reflection at the interpersonal level, as both experienced and novice trainers and apprentices can learn something by taking up another’s perspective. The participants in our study reported that networks are created under certain specific conditions—pre-existing networks, presence of highly committed individuals, small contexts, and problems with an apprentice. This facilitates the establishment of collaboration and can lead to transformation of practices.

Several elements emerged in the perspectives of stakeholders as potential boundary objects/crossers. Branch courses and practical courses at vocational schools are formally intended to connect school- and workplace-based learning, and the LPD and apprenticeship inspectors are mainly associated with workplace training. However, as per the perspectives (and expectations) of the stakeholders, they play a prominent role in connecting different practices. The literature on boundary objects argues that these should be “flexible in use and yet robust enough to keep their own identity across different practices. When too general […], they do not provide enough direction. When too specific […], they are not useful in different practices anymore” (Bakker and Akkerman 2019, p. 355). The LPD, for example, is not formally graded during the qualification procedures, which could explain why most learners and their in-company trainers are not motivated to use it (Caruso et al. 2016). In our data, several elements appear as boundary objects that can function as the learning mechanism of cooperation either at the interpersonal level (e.g., teachers and in-company trainers can coordinate by sharing their apprentices’ marks or exchanging plans and schedules at the beginning of the school year) or at the intrapersonal level (e.g., apprentices can align and make sense or their participatory positions in multiple practices through LPD or branch courses).

The degree of formality in the IVET partnership seems to be a recurrent topic in the stakeholders’ discourses. In fact, the balance between the aspects that should be formally established and those that should be left open to interpretation is extremely unclear. For example, to what extent are apprentices expected to guarantee the exchange of information between learning sites? To what extent can teachers or in-company trainers interfere in apprentices’ training? Or, to what extent should the LPD be used at both the training company and the school to connect the two practices? An attempt to strike a balance between formal and informal measures is found in the notion of compulsory and desirable collaboration (Peter 2014): finding a common base for compulsory collaboration to ensure a basic level of partnership, while also defining a desirable level of collaboration that does not restrict the freedom of all the stakeholders involved.

## Conclusion

Based on the results of this study, it seems that it is important to find a balance between the aspects that should be formally established in an IVET partnership and those that should be left as “desirable collaboration” (Peter 2014). Further, apprentices serving as brokers can potentially foster the connection between school and training company, but this role needs to be better defined and supported by their teachers and in-company trainers. In addition, networks that collaborate and promote an integrative view of IVET exist and provide examples of effective IVET partnerships. Their example could be promoted and could serve as a model for other contexts. These findings are consistent with the opinion of numerous scholars who stressed the need to develop mutual understanding among stakeholders to facilitate collaboration and better connect school with training company (Billett and Seddon 2004; Griffiths and Guile 2004; Sappa et al. 2018; Tynjälä 2008). According to this perspective, an approach that involves boundary crossing is interesting because it does not see boundaries as barriers to learning but as promotors of learning instead. The boundaries between the school and the workplace should not disappear: the epistemic nature of schools and training companies is not going to change and nor will the functions or expertise of vocational teachers and in-company trainers (Bakker and Akkerman 2019). However, to overcome the discontinuities created by these sociocultural differences, it is important that all the stakeholders involved develop boundary crossing awareness. Connecting school- and workplace-based learning is not the responsibility of apprentices only. The unit of analysis must be shifted from the individual apprentice to all the stakeholders involved in the IVET system and their respective institutions. Sappa et al. (2018) insist on the importance of the teachers and in-company trainers developing connective strategies. This entails that they need the ability and motivation to broaden their expertise and use strategies to connect school and workplace. Furthermore, numerous scholars have highlighted the importance of developing new work cultures (Bakker and Akkerman 2019; Deitmer and Heinemann 2009; Sappa et al. 2018; Tynjälä 2008), with “learning arrangements or interventions that do not treat school- and work-based learning as sequential and isolated, but rather as integrated and coherent” (Bakker and Akkerman 2019, p. 366). This change must also be implemented on a personal level, and not only at the pedagogical or organizational level (Sappa et al. 2018).

Finally, this study has certain limitations. The first is the fact that our questions were not directly about IVET partnership but, instead, about the perceptions of the quality of IVET. This allowed us to collect data in a more spontaneous manner and let elements emerge freely. However, those data could be enhanced by more direct questions and contextual elements (for example, collected through direct observation of practices). Second, we decided to consider only certain stakeholders on the ground without considering those at the meso (e.g., professional associations) and macro levels (e.g., state representatives) who also play a significant role in IVET partnerships. In future studies, it would be interesting to collect more specific data, including those obtained from stakeholders from various levels.

## Data Availability

The dataset generated and analysed during the current study are not publicly available due to confidentiality reasons. They are available from the author on reasonable request.

## Appendix

See Table

**Table 1:** Apprentices’, teachers’, and in-company trainers’ perspectives on the five themes of IVET partnership

		Apprentices	Teachers	In-company trainers
Degree of formality in IVET partnership	Formal contact	Useful for avoiding abuse, removing responsibility from apprentices, and receiving regular supervision vs Not good because you “can’t skip school,” less autonomy	In favor of more formal contact as long as it does not increase administrative work and does not interfere with teaching tasks	In favor of more formal contact as long as it does not increase administrative work and do not interfere with company operations
	Informal contact	–	Informal contact is considered good but difficult to establish and maintain. It is fragile Informal contact raises the questions “Who is supposed to initiate contact?” and “Why should I initiate this contact first?” Teachers tend to initiate contact in case there are problems with an apprentice	Informal contact is considered good but difficult to establish and maintain. It is considered fragile It raises the questions “Who is supposed to initiate contact?” and “Am I allowed to interfere in my apprentice’s life?” In-company trainers tend to initiate contact in case there are problems with an apprentice
Apprentices as brokers		–	Teachers tend to consider apprentices acting as brokers a good thing: They can create a connection between their school and training company, allowing the exchange or even expansion of practices. The school and training company can learn through the apprentices	Certain in-company trainers tend to think that apprentices who serve as brokers filter the information they communicate between their school and training company to suit their own needs
Teachers and in-company trainers as brokers		It is considered useful for teachers and in-company trainers to have hybrid profiles, especially in the case of teachers (“In class, they know what they are talking about.”)	It is considered useful for teachers and in-company trainers to have hybrid profiles, as teachers can stay updated with the evolution of their trade, it gives them credibility in the eyes of their apprentices, and they can discuss authentic situations in the classroom	It is considered useful for teachers and in-company trainers to have hybrid profiles Teachers with hybrid profiles can stay updated with the evolution of their trade
Boundary objects and other brokers	Branch courses	Useful because they do not have the constraints of school vs Not useful because they are repetitive and not aligned with the curricula used by the school or training company	–	–
	LPD	Useful for structuring, planning, evaluating, and monitoring the training vs Not useful because the process is repetitive or not aligned with the school and is time-consuming	–	–
	Apprenticeship inspectors	–	As per teachers, apprenticeship inspectors should ensure the establishment of a connection between schools and training companies Currently, inspections are carried out only in case there are problems with an apprentice	–
	Practical courses	Considered good but should be expanded and further linked to the training company	–	–
Examples of networks		–	Example 1: Hairdressing teachers explained that, in their state, they are used to working with other stakeholders (training companies, professional associations, regional authorities, and apprentices’ families) and that they regularly organize joint activities	Example 2: The group of in-company trainers from the construction field had an integrative vision regarding IVET. They held hybrid profiles and worked regularly in collaboration with the other learning locations Example 3: A commercial employee in-company trainer mentioned the creation of a network between novices and experienced in-company trainers and apprentices with the aim of learning from each other

1.

## Abbreviations

IVET: Initial vocational education and training
VET/PET: Vocational education and training/Professional education and training
LPD: Learning and performance documentation
SERI: State Secretariat for Education, Research and Innovation

## Footnotes

1 Branch courses are usually organized by professional associations in specific training centers for one to three weeks per year. They are designed in order to provide apprentices with the theoretical and practical basics of the trade. Therefore, they group together apprentices from the same trade but from several companies and regions (Wettstein et al. 2014).

2 More details about the project can be found here: https://www.sfivet.swiss/project/quality-initial-vet.

3 The code was named as per the language used by the participants to the focus groups.

4 All excerpts mentioned in this section were originally in French. The author translated and slightly adapted them for a better intelligibility.

5 Apprenticeship inspectors are in charge of monitoring the quality of training, primarily at the workplace. They verify whether a training company fulfils the training requirements and monitor the training provided at the workplace through meetings with the apprentice and the in-company trainer. They are engaged either by the state or by the professional association and often have “hybrid” profiles. Many of them are professionals who still work in the field part-time.
